# A Portable UV-LED/RGB Sensor for Real-Time Bacteriological Water Quality Monitoring Using ML-Based MPN Estimation

**DOI:** 10.3390/bios15050284

**Published:** 2025-04-30

**Authors:** Andrés Saavedra-Ruiz, Pedro J. Resto-Irizarry

**Affiliations:** 1 Bioengineering Graduate Program, University of Puerto Rico Mayagüez, Mayagüez, PR 00680, USA; andres.saavedra@upr.edu; 2 Mechanical Engineering Department, University of Puerto Rico Mayagüez, Mayagüez, PR 00680, USA

**Keywords:** water quality (WQ), defined substrate test (DST), most probable number (MPN), machine learning (ML), point-of-care

## Abstract

Bacteriological water quality monitoring is of utmost importance for safeguarding public health against waterborne diseases. Traditional methods such as membrane filtration (MF), multiple tube fermentation (MTF), and enzyme-based assays are effective in detecting fecal contamination indicators, but their time-consuming nature and reliance on specialized equipment and personnel pose significant limitations. This paper introduces a novel, portable, and cost-effective UV-LED/RGB water quality sensor that overcomes these challenges. The system is composed of a multi-well self-loading microfluidic device for sample-preparation-free analysis, RGB sensors for data acquisition, UV-LEDs for excitation, and a portable incubation system. Commercially available defined substrate technology, most probable number (MPN) analysis, and machine learning (ML) are combined for the real-time monitoring of bacteria colony-forming units (CFU) in a water sample. Fluorescence signals from individual wells are captured by the RGB sensors and analyzed using Multilayer Perceptron Neural Network (MLPNN) and Support Vector Machine (SVM) algorithms, which can quickly determine if individual wells will be positive or negative by the end of a 24 h period. The novel combination of ML and MPN analysis was shown to predict in 30 min the bacterial concentration of a water sample with a minimum prediction accuracy of 84%.

## 1. Introduction

Waterborne diseases pose a significant threat to public health, leading to an annual occurrence of 7.15 million cases and 6630 deaths in the United States alone [1]. This includes diseases such as cholera, typhoid fever, hepatitis, gastrointestinal ailments, and respiratory tract infections, often originating from pathogenic microorganisms such as bacteria, viruses, fungi, and intestinal worms. As a result, monitoring water quality is indispensable for safeguarding public health and preserving ecological equilibrium [2,3].

Effective water quality analysis depends on measuring various parameters. While traditional physico-chemical indicators like the potential of hydrogen (pH), electrical conductivity (EC), turbidity (Turb), total dissolved solids (TDS), total phosphorus (TPh), total nitrogen (TNit), and dissolved oxygen (DO) offer insights into water quality [4], they do not directly reflect potential health or agricultural implications. The detection of bacteria, including total coliforms, *Escherichia coli* (*E. coli*), and *Enterococcus faecalis* (*E. faecalis*), is one of the most used metrics for assessing bacteriological contamination [5]. These indicators, characterized by distinct biochemical traits, serve as reliable markers to determine the suitability of water and food for human consumption [6]. The geometric mean (GM) and statistical threshold value (STV), measured in colony-forming units (CFU) per 100 mL, are the statistical values used in bacteriological water quality standards. The 2012 Recreational Water Quality Criteria (2012 RWQC) recommends thresholds of 35 CFU per 100 mL GM for marine and freshwater enterococci and 126 CFU per 100 mL GM for *E. coli* in freshwater [7]. Similarly, the Beach Action Value (BAV) defines limits of 70 CFU per 100 mL for marine and freshwater enterococci and 235 CFU per 100 mL for *E. coli* in freshwater [7].

Established methods such as multitube fermentation (MTF) and membrane filtration (MF) are often viewed as the gold standard for assessing water quality. These techniques are supported by standardized and accredited protocols that facilitate inter-laboratory comparisons. However, they present drawbacks, including the need for cold transportation, trained personnel, laboratory space and equipment, expensive consumables, and a minimum 24 h incubation period for obtaining results. Alternative methods include molecular and enzyme-based techniques, which mitigate some of these drawbacks and offer significantly faster detection [8,9]. The defined substrate technology (DST) method, exemplified by Colilert^TM^ and Enterolert^TM^ (IDEXX Laboratories, Inc., Westbrook, ME, USA), uses enzyme-substrate fluorescence to quantify total coliforms, *E. coli*, and enterococci [10,11]. This EPA-approved approach is easier to use than traditional methods and features a streamlined process, although it may involve human error during quantification. The IDEXX Quanti-Tray/2000 (IDEXX Laboratories, Inc., Westbrook, ME, USA) divides a 100 mL sample into 97 wells of two different sizes and uses the most probable number (MPN) method to determine the bacterial count in the original sample [11]. However, like MTF and MF, DST requires specialized laboratory equipment and cold transportation, and it has an incubation time of 24 h per test.

Recent advancements in sensor technology have introduced alternative methods to accelerate bacterial detection. For instance, the Colifast ALARM^TM^, developed by the Norwegian company Colifast (Colifast AS, Lysaker, Norway), automates detection and reduces the time to detection by using DST and focusing on the presence of fluorescence due to bacterial growth. The system collects and incubates water samples in a specialized chamber connected to a detector. The substrate 4-methylumbelliferyl-β-D-glucuronide is used for detecting *E. coli*, while 4-methylumbelliferyl-β-D-galactoside is used for detecting total coliforms [12,13]. However, a disadvantage of this system is that it requires installation in a fixed setting, limiting its use in field applications and potentially increasing the operational costs due to the need for dedicated space and technical oversight. Additionally, the company has developed the Colifast Field Kit, which enables measurements within 75 min to 2 h and is more cost-effective than the standard version. However, its detection limits are tailored to concentrations above 500 CFU per 100 mL. However convenient, many of these systems require investments between thousands to tens of thousands of dollars. These systems use ultraviolet (UV) light and fluorescence as indicators of the presence of bacteria.

Ultraviolet and color light-emitting diodes (LEDs) and RGB sensors can serve as low-cost alternatives to traditional water quality monitoring equipment. UV light has ubiquitous applications in bioengineering, including sterilization, phototherapy, the activation of photosensitive compounds, and microscopy. In the field of water quality, ultraviolet–visible (UV-Vis) spectroscopy has been used to measure parameters such as chemical oxygen demand, heavy metal ions, nitrate-nitrogen, and dissolved organic carbon. These precise measurements are essential to ensure the safety of drinking water and preserve ecological balance [14]. Other examples include the combination of UV-Vis and fluorescence spectroscopy to enable real-time detection of biological and chemical contaminants [15]. Zhang et al. employed UV-Vis spectrometry combined with artificial neural networks to evaluate key water quality parameters, including total organic carbon (TOC), total suspended solids (TSS), and chemical oxygen demand (COD), underscoring its potential for real-time water quality assessment [16]. Lyu and Zhao present the development of statistical regression models and artificial neural networks to estimate concentrations of nitrogen, phosphorus, COD, and suspended solids in eutrophic rivers using UV-Vis spectroscopy, concluding that these models demonstrate a high level of accuracy and reliability in predicting water quality parameters [17].

RGB sensors have been applied to monitor different parameters related to water quality. Parra et al. developed, calibrated, and tested a low-cost optical sensor for measuring and classifying water turbidity using an RGB LED in combination with a photoresistor. The data collected were then analyzed using machine learning models determining turbidity with an accuracy of 91.23% when employing the K-Nearest Neighbor algorithm [18]. Similarly, Sampedro and Salgueiro incorporate red, green, blue, and infrared light sources with a photoresistor to enable remote turbidity measurements in aquatic environments without the need for complex analytical equipment [19]. Benavides et al. demonstrated the use of low-cost RGB sensors for monitoring microalgae growth in a photobioreactor. The sensor was calibrated using a commercial spectrophotometer for offline measurements and a submerged electrode for real-time, online monitoring. This research highlights the significance of monitoring and process control in microalgae production, with applications extending to biofuels, pigments, cosmetics, animal feed, and wastewater treatment [20]. Pazzi et al. used TCS3200 RGB sensors with an Arduino microcontroller and microfluidic paper-based analytical devices (µPADs) for measuring pH, showing high accuracy and alignment with values obtained from a traditional pH meter [21]. In contrast to the scientific literature, the approach presented in this manuscript is, to the author’s knowledge, the first to integrate UV excitation LEDs and RGB sensors in an acrylic microfluidic device for bacteriological water quality measurements.

Microfluidic devices can be integrated with excitation LEDs and fluorescence sensors and can offer low-cost, portable, and automated capture and analysis of small volumes. Lab-on-a-chip sensors have been developed for water quality monitoring, specifically for detecting microorganisms, heavy metals, organic compounds, and various other substances [22]. Recent research has led to the development of microfluidic-based technologies that enable the monitoring of multiple water contaminants with precision comparable to traditional methods while significantly reducing detection times. In one such study, Gowda et al. designed a microfluidic device capable of capturing water samples, lysing bacterial cells, extracting nucleic acids, and quantifying bacteria using a droplet digital loop-mediated isothermal amplification assay. This portable system can quickly detect bacteria like *E. faecalis* directly on-site, completing the entire test in just 1 h and requiring less than 5 min of manual sample handling [23].

Additional innovations aimed at reducing the detection time for *E. faecalis* have included recombinase polymerase amplification (RPA) combined with a lateral flow assay (LFA). This method allows for testing within approximately 30 min, a significant reduction compared to the 24 h required by the Enterolert™ (IDEXX Laboratories, Inc., Westbrook, ME, USA) test. However, the RPA-LFA approach compromises sensitivity, detecting *E. faecalis* with 10 to 1000 times lower efficiency compared to Enterolert™ [24]. This highlights the ongoing challenge of developing low-cost technologies that are both rapid and sensitive enough for effective water quality monitoring.

To accelerate the detection of bacteria in water, machine learning (ML) can be used with lab-on-a-chip concepts to efficiently capture, monitor, and store water samples. As a branch of artificial intelligence (AI), ML involves creating algorithms that enable computers to learn from data and enhance their performance over time [25,26]. By developing mathematical models that identify patterns and relationships within datasets, ML can predict the behavior of new data, making it an effective tool for real-time water quality monitoring. Paepae et al. examined the most used parameters and machine learning (ML) algorithms in water quality research. They identified four predominant algorithms: neural networks (NN), random forest (RF), multiple linear regression (MLR), and support vector machines (SVM) [4]. Neural networks were favored for their ability to model complex nonlinear relationships in water quality data, making them highly suitable for tasks requiring high predictive accuracy [27]. Random Forest gained popularity for its robustness, capacity to handle large datasets with high dimensionality, and effectiveness in reducing overfitting [28,29]. The multiple linear regression (MLR) facilitates hypothesis testing and model selection. To enhance reliability and consistency, steps such as multicollinearity diagnostics, cross-validation, and regularization are conducted prior to model development. In water quality index (WQI) modeling, MLR integrates multiple physicochemical parameters into a single score that represents overall water quality [30]. The Support Vector Machine (SVM) algorithm works well with small datasets but can face computational efficiency issues with larger ones. Conversely, limited sample sizes may not sufficiently capture classification features. Effective data streamlining is essential to preserve support vectors and maintain data integrity [31].

In this study, rapid prototyping and open-source tools are used to create a water quality sensor with a microfluidic device and everything necessary to carry out and quantify a DST assay at the point-of-care. MPN quantification occurs by observing the number of positive (fluorescing) wells in the microfluidic device. Each well is loaded with the Enterolert™ substrate mixed with bacteria-containing water and placed within a portable incubation system. The system uses UV light excitation and RGB sensors to monitor the water samples in each well, generating a dataset to train two machine learning algorithms: multilayer perceptron neural networks (MLPNN) and support vector machine (SVM). Once trained, the ML can quickly predict whether a well will be positive or negative after the 24 h period. In conjunction with the MPN table, this technique predicts the water sample MPN in a few hours using low-cost, open-source materials.

## 2. Materials and Methods

### 2.1. Microfluidic Device Design and Loading

**Figure 1:**
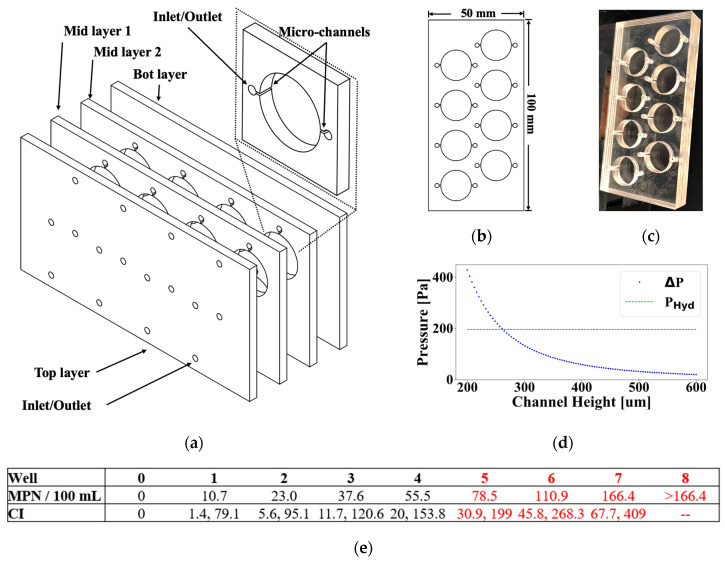
(**a**) Microfluidic device configuration. (**b**) Dimensions and structure of acrylic sheets. (**c**) Fabricated microfluidic device. (**d**) Hydrostatic versus hydraulic resistance pressure analysis. (**e**) MPN table and CI of microfluidic device; red values indicate contamination beyond EPA BAV threshold.

The microfluidic device was designed to facilitate both sample handling and the most probable number (MPN) quantification of bacteria. The microfluidic device was constructed from four acrylic sheets, including a base sheet without apertures, two intermediate sheets, each featuring eight wells with channels for fluid movement, and a top sheet solely for fluid inlets/outlets (Figure 1a). The dimensions of each acrylic sheet measured 50 mm × 100 mm, and the wells possessed a diameter of 16 mm (Figure 1b). Inlet and outlet ports connect to each well through channels of 500 µm height, *h*; 3.175 mm width, *w*; and 22 mm length, *L*, respectively. *L* was taken as the distance between the inlet and outlet ports. The device was fabricated using 3.175 mm cast acrylic sheets and an Epilog 60W CO_2_ laser cutter (Figure 1c). The CO_2_ laser cutter operated at 25% speed and 85% power. Double-sided medical-grade adhesive (ARcare^®^ 90106, Adhesives Research, Inc., Glen Rock, PA, USA) was used to bond the four acrylic layers. The microfluidic device was designed to fill with liquid passively by comparing the approximate hydraulic resistance of the channels ${(R}_{Hyd}=(12\eta L)/(1-0.63\left(\frac{h}{w}\right)h^{3}w)$ to the hydrostatic pressure ${(P}_{Hyd}=\rho gL)$ provided at the channel inlet when submerged. A comparison is shown in Figure 1d where *P_Hyd_* is the hydrostatic inlet pressure, and ∆*P* represents the pressure necessary to overcome the hydraulic resistance of the channel, $∆P=QR_{Hyd}$, assuming a volumetric flow rate Q of 5 mL/min. The analysis demonstrated that for the selected dimensions (*L* = 22 mm, *w* = 3.175 mm), a channel height *h* above 280 μm results in efficient filling, while heights below led to incomplete filling due to a lack of inlet pressure. For the designed system, each well requires 1.25 mL of sample water, taking a few seconds to fill completely, with a channel height of 500 μm. Figure 1e displays the MPN table and confidence intervals for the microfluidic device using Equations (1) and (2), obtained from Jarvis et al. [32], where $t_{p}$ refers to the number of positive tubes, $t_{T}$ is the total number of tubes (8 wells were used in this case), det is the dilution level per tube (1 when utilizing the original concentration of the water sample), and $v_{et}$ is volume per tube (1.25 mL). Additionally, Equation (2) was used to calculate the confidence limits (${CI}_{limit}$), where σ represents the standard deviation associated with each MPN value, *d*.

(1) $$MPN=-\frac{Ln\left(1-\frac{t_{p}}{t_{T}}\right)}{(det)v_{et}}$$

(2) $${CI}_{limit}=\left[de^{-2\sigma_{\ln\left(d\right)}},de^{2\sigma_{ln(d)}}\right]$$

The volume and number of wells were chosen to include the EPA BAV threshold of 70 CFU, which is the limit used to determine if water is suitable for recreational activities. The detection range, when all wells are positive, is 166.4 CFU/100 mL. The detection range and resolution could be tailored for different applications and involve changing the number of wells and volume per well.

In the experiments, 108 mL of distilled water and a lyophilized *E. faecalis* pellet (*Enterococcus faecalis* WDCM Vitroids™, Sigma-Aldrich Co. LLC, St. Louis, MO, USA) hydrated in 2 mL of PBS were used. The IDEXX Enterolert™ (IDEXX Laboratories, Inc., Westbrook, ME, USA) reagent was then added to the 110 mL solution and was thoroughly shaken to ensure homogeneity. The microfluidic device was filled with 10 mL of this mix, while 100 mL was used to fill the IDEXX Quanti-Tray/2000. The inlets and outlets of the microfluidic device were sealed with polyester film PET tape and incubated for 24 h in a portable platform. The Quanti-Tray/2000 was incubated for 24 h in an incubator oven at 41 °C.

### 2.2. Portable Temperature-Controlled Incubation and UV-LED/RGB Excitation and Emission System for Bacterial Detection

**Figure 2:**
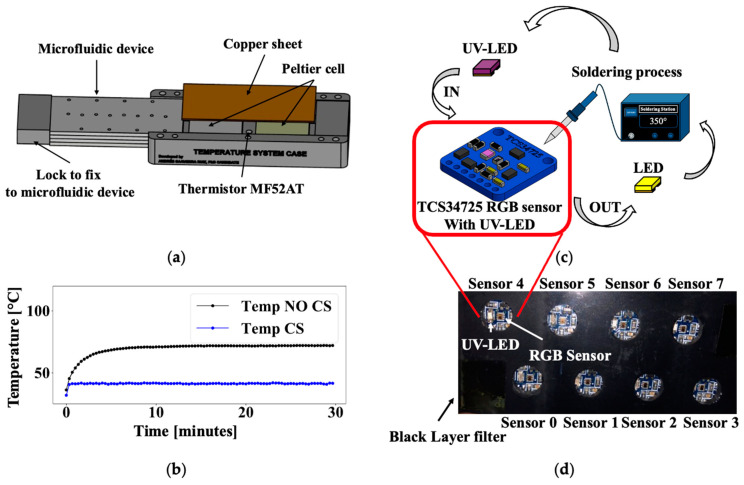
(**a**) Case for Peltier cell and microfluidic device. (**b**) Temperature response with and without control system (CS). (**c**) RGB sensor with UV-LED to detect fluorescence in water samples. (**d**) Array of 8 UV-LED/RGB sensors with a black layer filter.

Achieving the required incubation temperature of 41 °C for a 24 h period is vital for accurate results. A temperature control system was developed using a Peltier thermoelectric cooler (Digi-key Electronics, TEC-12706, Thief River Falls, MN, USA) as the heating element, a copper sheet to distribute heat, and a thermistor (Einstronic, MF52AT, Kota Kinabalu, Malaysia) as the temperature sensor. These components are controlled by an Arduino Nano 33 IoT (Digi-key Electronics, part ABX00027, MN, USA), which interfaces with the Peltier cell via a motor drive module (Einstronic, MX1508, Kota Kinabalu, Malaysia). A customized case was designed in Solid Works and fabricated using a 3D printer (Creality CR 10S, Shenzhen, China) and holds the Peltier cell, copper sheet, and microfluidic device, Figure 2a. Figure 2b presents the temperature response of the system with and without a control system (CS), demonstrating the effectiveness of the control system in stabilizing temperature fluctuations.

The UV-LED/RGB system combines RGB sensors (Adafruit Industries LLC, TCS34725, New York, NY, USA) with 365 nm wavelength UV LEDs (Digi-key Electronics, part 1497-1479-1-ND, MN, USA), all controlled by the Arduino Nano 33 IoT. Originally designed with an LED emitter, the RGB sensors were calibrated using various color sheets (white, black, red, green, and blue). UV LEDs are then integrated into the RGB sensor system to enable fluorescence detection in water samples, Figure 2c. The combination of the RGB sensors and UV-LEDs is named the UV-LED/RGB system. To enhance accuracy and minimize noise, a black acrylic layer is incorporated into the RGB system as a filter, effectively reducing UV light scattering (Figure 2d).

**Figure 3:**
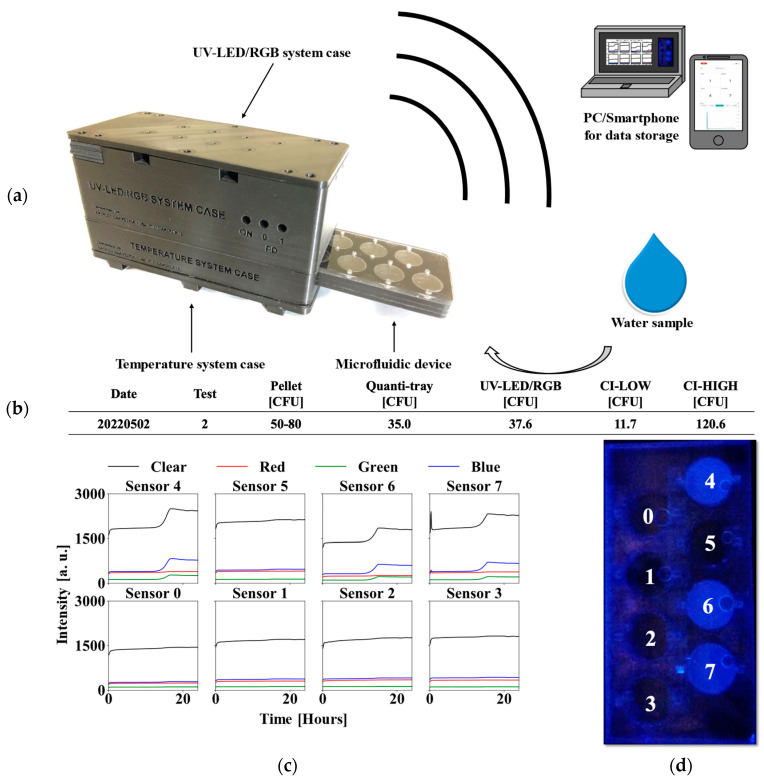
(**a**) The UV-LED/RGB system integrates a microfluidic device containing a water sample and transmits data monitored by the RGB sensors to a computer or smartphone, either through a wired or wireless connection. (**b**) Summary of results from 50 to 80 CFU pellet experimental test. (**c**) RGBC data from UV-LED/RGB system. (**d**) Microfluidic device exposed to UV light and fluorescence in wells 4, 6, and 7. The other wells (0–3 and 5) indicate negative for bacteria.

The UV-LED/RGB system is designed with a temperature control unit at the base, where the microfluidic device is inserted. The RGB sensor monitoring system is positioned in the upper section, Figure 3a. These RGB sensors communicate with the microcontroller using I2C communication protocol, with a multiplexer (Texas Instruments, TCA9548A, Dallas, TX, USA) enabling simultaneous communication with up to eight RGB sensors. Data could be viewed in real time through either wired or wireless connections. A comprehensive database was constructed, incorporating clear, red, green, and blue signals for each sensor, yielding a total of thirty-two columns of data used as inputs in the machine learning algorithms.

Figure 3b shows a summary of results from an experiment using a water sample inoculated with a 50–80 CFU pellet. The IDEXX Quanti-Tray/2000 was used as a benchmark and measured a bacterial concentration of 35 CFU/100 mL, while the UV-LED/RGB system measured 37.6 CFU/100 mL, using the same water sample and the custom-made MPN table (Figure 1e). Figure 3c shows red, green, blue, and clear (RGBC) channel data for individual wells in the microfluidic device. It is important to note that bacterial quantification can be performed by counting the number of positive wells after 24 h, but this time-to-detection can be greatly reduced by studying the interactions between the RGBC curves in real-time during incubation. Data were acquired every 20 s, resulting in 4320 observations in a 24 h incubation period. Figure 3d shows a picture of the microfluidic device when exposed to UV light. The device’s wells are numbered from 0 to 7. Fluorescence appears in wells 4, 6, and 7, indicating a CFU of 37.6 CFU according to Figure 1e. This fluorescence corresponds to the exponential growth curves in Figure 3c at approximately hour 15 for wells 4, 6, and 7 and serves to confirm the growth detected by the UV-LED/RGB system.

For this study, 11 experimental tests were conducted, distributed as follows: 4 tests with concentrations ranging from 50 to 80 CFU, 3 tests from 80 to 120 CFU, 1 test from 130 to 300 CFU, 1 test from 3k to 7k CFU (undiluted), 1 test from 50k to 150k CFU (undiluted), and 1 test from 50k to 150k CFU (diluted 1:1000).

### 2.3. Machine Learning (ML) Algorithms

Two machine learning (ML) algorithms, support vector machine (SVM) and multilayer perceptron neural networks (MLPNN), were selected for this study due to their widespread application in water quality analysis. Equation (3) applies the first derivative to the RGB sensor signals, denoted as *c* for clear, *r* for red, *g* for green, and *b* for blue. This transformation is incorporated into the dataset used by machine learning algorithms to enhance their performance.

(3) $$\frac{\partial c_{\left(t\right)}}{dt},\;\frac{\partial r_{\left(t\right)}}{dt},\;\frac{\partial g_{\left(t\right)}}{dt},\;\frac{\partial b_{\left(t\right)}}{dt}$$

**Figure 4:**
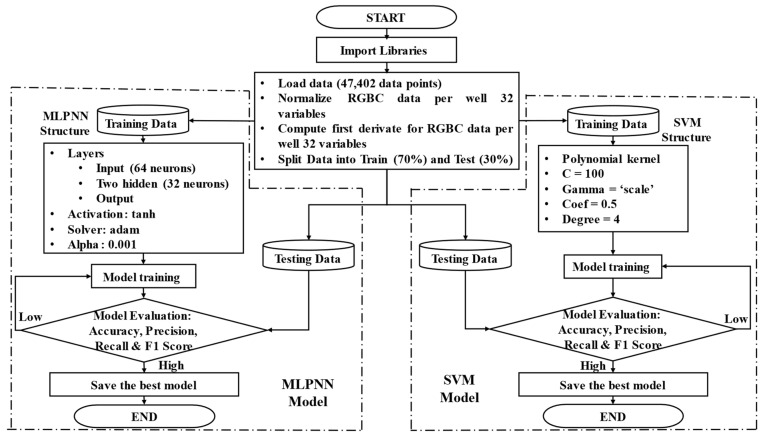
Machine learning flowchart: The left side depicts the MLPNN algorithm, while the right side outlines the SVM algorithm.

The SVM algorithm was developed in Python version 3.8.8, an open-source software, using a combination of ML and data manipulation libraries such as NumPy, Pandas, Scikit-Learn, TensorFlow, and Keras. A normalized and derivative-transformed dataset (using Equation (3)) includes measurements from 8 UV-LED/RGB sensors, with inputs for each sensor representing values for clear, red, green, and blue, both in their original and derived forms. The dataset is divided into training and testing sets with a 70:30 ratio using Scikit-Learn’s train_test_split function. The SVM model is fitted with a polynomial kernel, with tuned parameters (C = 100, gamma = ‘scale’, coef0 = 0.5, degree = 4), to capture nonlinear relationships in the data. The model’s performance is evaluated using metrics such as accuracy, precision, recall, and F1-score, represented by Equations (4)–(7) [33], where TP stands for true positives, TN for true negatives, FP for false positives, and FN for false negatives. These metrics are visualized for each sensor using bar graphs, and the results are plotted using Matplotlib version 3.7.5 to generate detailed graphs of each sensor’s performance. The flowchart of the SVM algorithm is shown on the right-hand side of Figure 4.

Similarly, the MLPNN algorithm was implemented in Python, leveraging TensorFlow and Keras to build and train the neural network model. The data preprocessing steps involved loading normalized and derivative-transformed datasets, followed by splitting the data into training and testing sets. The neural network architecture consisted of a sequential model with multiple dense layers, configured with 32 neurons in each hidden layer (2) and using the ‘tanh’ activation function to capture complex relationships between input features. The model was optimized using the ‘adam’ solver and an adaptive learning rate, with regularization parameter alpha set to 0.001, and the maximum number of iterations increased to 1000 to ensure convergence. As with SVM, the performance of the MLPNN model was evaluated using metrics such as accuracy, precision, recall, and F1-score (Equations (4)–(7), respectively) [33], which are visualized for each sensor using bar graphs, ensuring robust model development. A plotting stage was also included to visualize the predicted and actual values, demonstrating the model’s ability to generalize across different datasets. The flowchart of the MLPNN algorithm is shown on the left-hand side of Figure 4.

(4) $$Accuracy=\frac{TP+TN}{TP+TN+FP+FN}$$

(5) $$Precision=\frac{TP}{TP+FP}$$

(6) $$Recall=\frac{TP}{TP+FN}$$

(7) $$F1\;Score=2\left(\frac{\left(Precision)(Recall\right)}{Precision+Recall}\right)$$

## 3. Results and Discussion

### 3.1. UV-LED/RGB System

**Figure 5:**
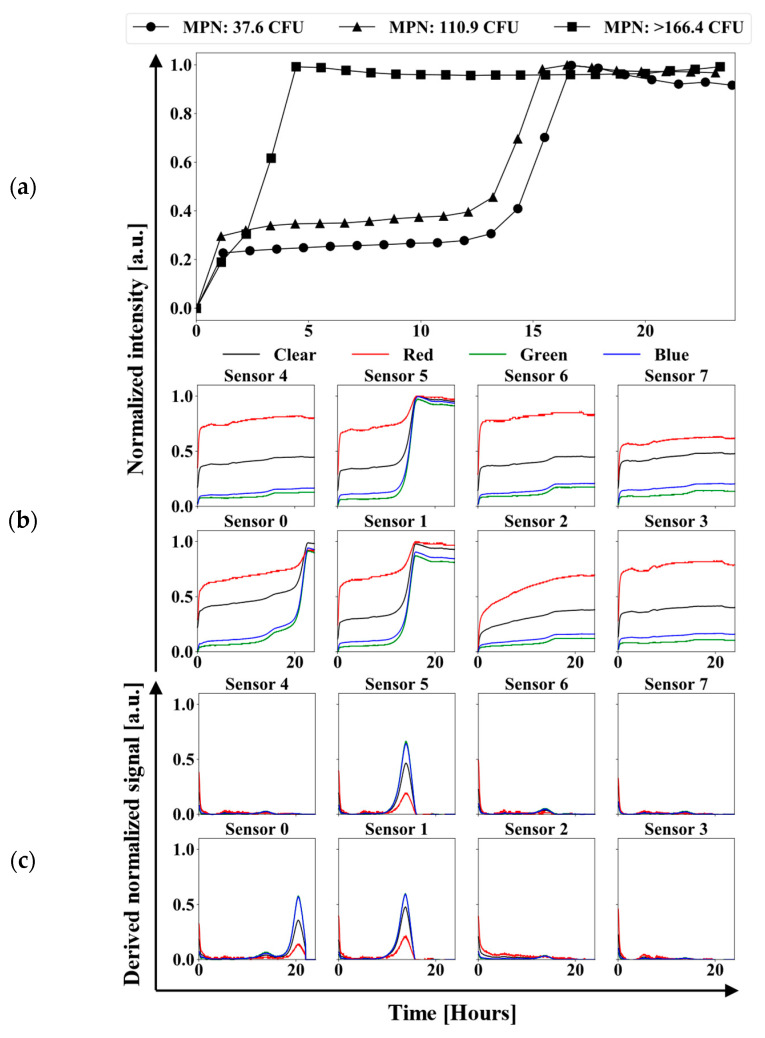
(**a**) UV-LED/RGB sensor number 4 “clear” color channel showing sigmoidal bacteria growth curves for different concentrations of bacteria in water samples. (**b**) Normalized RGB signals from the experiment measuring 37.6 CFU/100 mL. (**c**) First derivative curves from 37.6 CFU/100 mL experiment.

Several experiments were conducted using the UV-LED/RGB system, comparing its results with those obtained from the IDEXX Quanti-Tray/2000 system. The experiments were carried out using different concentrations of bacteria to assess the performance of the UV-LED/RGB system. Figure 5a shows “clear” color channel signals from sensor number 4 for experiments with different bacterial concentrations: 37.6, 110.9, and >166.4 CFU/100 mL. All curves exhibit the characteristic sigmoidal behavior of bacterial growth, confirming the system’s ability to monitor bacterial growth in real time. It can be observed that as the bacterial concentration increases, the growth curve’s slope becomes steeper, and the exponential phase begins earlier.

Figure 5b illustrates the normalized RGBC signals from an experiment with a bacterial concentration of 37.6 CFU. Wells 0, 1, and 5 demonstrated a positive response to the presence of bacteria, as indicated by their sigmoidal trends throughout the incubation period, unlike the other wells. This figure provides a clear example of monitoring low bacterial concentrations in water, adhering to the EPA BAV standards for recreational activities (37.6 CFU/100 mL). The exponential phase for wells 1 and 5 occurs at approximately 12 h into the incubation period, suggesting that this duration is necessary to detect bacterial presence. In contrast, well 0 required more than 20 h to reach the exponential phase, highlighting that solely interpreting sensor signals might not be sufficient for accurately determining bacterial concentrations in water samples within the first 12 h.

Figure 5c presents the first derivative of the normalized signals from the same experiment, highlighting the same wells, 0, 1, and 5, as positive. Applying the first derivative to the normalized data serves as an alternative method to determine contamination in wells. It also helps in reducing the detection time of bacterial growth in the wells because the positive slope of the exponential phase first derivative can easily be distinguished from the flat curves of non-fluorescing wells. The first derivative data also provides additional information to the ML algorithms for predicting wells with bacterial contamination.

The UV-LED/RGB system was benchmarked against the IDEXX Quanti-Tray/2000 using lyophilized pellets of different CFU concentrations, as shown in Table 1. The UV-LED/RGB system presents a smaller detection range of 10.7 to 166.4 CFU versus the Quanti-Tray/2000’s range of 1 to 2419 CFU. The UV-LED/RGB system showed lower accuracy, with an average of 55.9 CFU (STD 31.7 CFU) when using 50–80 CFU pellets, compared to 37.2 CFU (STD 4.0 CFU) for the Quanti-Tray/2000. The UV-LED/RGB and Quanti-Tray/2000 systems detected averages of 81.6 CFU (STD 64.4 CFU) and 82.1 CFU (STD 8.5 CFU) when using 80–120 CFU pellets, respectively. When using 130–300 CFU pellets, the UV-LED/RGB system reported 110.9 CFU versus 165.8 CFU for the Quanti-Tray/2000. Both systems accurately detected concentrations exceeding their upper detection limits. When using 3–7K CFU and 50–150K CFU pellets, the UV-LED/RGB reported >166.4 CFU, while the Quanti-Tray/2000 reported >2419.6 CFU. Overall, results from the UV-LED/RGB system are consistent with the Quanti-Tray/2000, but it suffers from larger margins of error than the benchmark. The measurements obtained from the UV-LED/RGB may not always be the same as the Quanti-Tray/2000, but the reference results fall within the confidence intervals shown in Figure 1e.

### 3.2. Machine Learning Algorithms

The database for the ML algorithms is divided into 70% for the training stage and 30% for the testing stage. Initially, the full 24 h of data from each experiment are used to develop the model. The database for the 24 h period consists of 47,402 data points, with 33,181 (70%) allocated for the training stage and 14,221 (30%) for the testing stage. During the training stage, the output variable, or target, is defined by assigning a “0” for the absence of bacteria in the well and a “1” for the presence of bacteria. Subsequently, the database is restricted to the lag phase in the experimental data, allowing the ML algorithms to analyze this phase with the aim of reducing the detection time.

**Figure 6:**
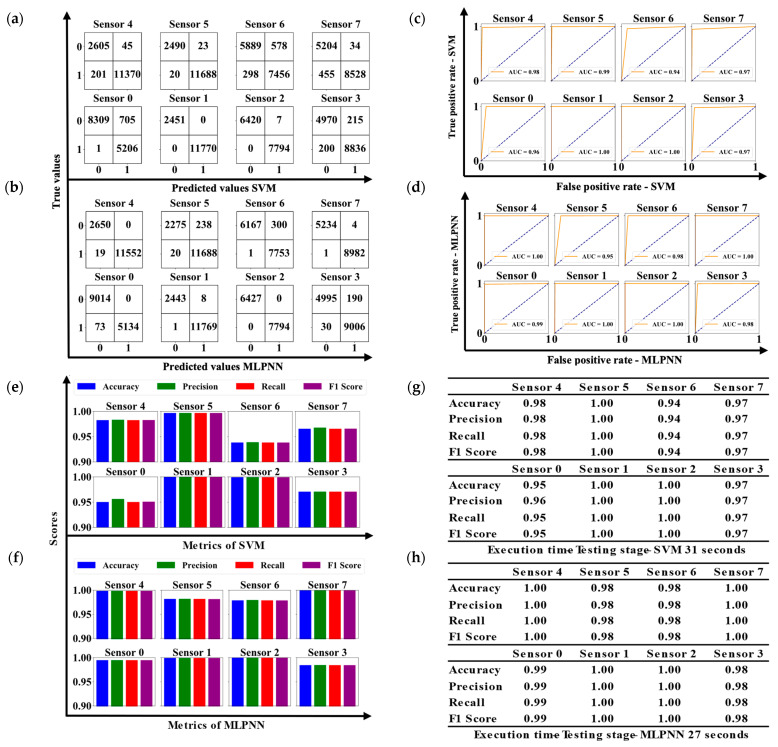
(**a**,**b**) Confusion matrix for each well of the UV-LED/RGB system after applying the SVM and MLPNN algorithm, respectively. (**c**,**d**) ROC curves with the AUC value for each SVM and MLPNN model used on the data acquired from each well. (**e**,**f**) Metrics of accuracy, precision, recall, and F1-score for the SVM and MLPNN models. (**g**,**h**) The metrics table for each well, along with the execution time for each ML algorithm.

Figure 6a,b illustrates the confusion matrix for each sensor when using the SVM and MLPNN algorithms, respectively, providing a visual representation of performance in terms of correct and incorrect classifications. The receiver operating characteristic (ROC) curves for both models are presented in Figure 6c,d, highlighting each algorithm’s discriminative capability through their respective areas under the curve (AUC). The graphs comparing accuracy, precision, recall, and F1 score metrics between the algorithms are displayed in Figure 6e,f, emphasizing the performance differences between SVM and MLPNN. Figure 6g,h presents the metrics in tabular form alongside the execution times for each algorithm, offering a concise and comparative summary of their efficiency and effectiveness.

Upon analyzing Figure 6g,h, it becomes evident that the performance of the MLPNN and SVM algorithms was evaluated across several sensors, with both demonstrating high effectiveness in data classification, consistently achieving strong metrics in accuracy, precision, recall, and F1 score. However, the comparative analysis reveals subtle differences between the two models. MLPNN exhibited more consistent performance, achieving perfect or near-perfect metrics across most sensors, including sensors 4, 6, and 0, where it achieved exceptional results with values of 1.00 or 0.99 in all metrics. On the other hand, SVM also showed excellent performance, achieving perfect metrics in sensors 5, 1, and 2. Nevertheless, it experienced a slight decline in performance on other sensors, particularly on Sensor 6, where the metrics dropped to 0.94. Sensors 7 and 3 exhibited slightly lower metrics with the SVM algorithm, reaching values of 0.97. This variability suggests that while SVM is robust, it may face more challenges in correctly classifying in certain specific contexts or may require further fine-tuning of its hyperparameters or data preprocessing techniques to match MLPNN’s consistency.

In terms of efficiency, MLPNN completed its execution in 27 s, while SVM took 31 s. Although both times were relatively quick, MLPNN was not only more consistent in its performance but also slightly more efficient in terms of execution time. These results suggest that MLPNN may better handle certain data patterns and offer more robust and efficient classification compared to SVM in this set of tests.

The UV-LED/RGB was shown to predict bacteria concentration in as little as half an hour by analyzing the lag phase of the sigmoidal curve. It was previously shown that the system quantifies bacteria concentration using the MPN method and counting how many wells are positive at the end of the 24 h incubation period. Predicting the bacteria concentration was performed by using trained MLPNN and SVM algorithms that predicted which individual wells would be positive by the end of the 24 h period using only data from the lag phase. Table 2 shows the performance of each algorithm using data from different periods of the lag phase (3, 2, 1, and 0.5 h) and highlights the performance of MLPNN and SVM for concentration prediction. The database was distributed accordingly: 3 h (5940 total data points: 4158 for training and 1782 for testing), 2 h (3960 data points: 2772 for training and 1188 for testing), 1 h (1980 data points: 1386 for training and 594 for testing), and 30 min (990 data points: 693 for training and 297 for testing). The experiments showed that it is crucial that each ML algorithm is trained with data from each lag phase period analyzed. The prediction ultimately centers on the interaction between the signal colors and the first derivative of each signal for the different periods of the lag phase.

While MLPNN excelled with nearly perfect metrics in sensors 0, 1, and 2 during longer latency phases (3 h), its performance declined notably as the latency period shortened, particularly in sensors 3, 6, and 7, where accuracy dropped as low as 0.72 at 0.5 h. This suggests that MLPNN struggles to maintain consistency in scenarios requiring faster response times, likely due to the complexity of the temporal data patterns. In contrast, SVM demonstrated greater robustness across all latency phases, achieving consistently high accuracy even in shorter latency windows. For example, while MLPNN saw a marked reduction in performance at 0.5 h, SVM maintained relatively stable metrics, only showing slight drops in sensors 2, 3, and 5. This resilience in SVM suggests that, although slightly less efficient in terms of execution time, it may provide more reliable performance in real-time or near-real-time applications where shorter latency periods are critical. Therefore, while MLPNN offers superior classification in more stable, extended time frames, SVM proves to be a more versatile option under dynamic temporal constraints.

## 4. Conclusions

Experiments with the UV-LED/RGB system demonstrate its strong potential for real-time bacterial growth monitoring in water samples at the point of care. The microfluidic device and MPN approach yielded consistent results when comparing the results to the benchmark Quanti-Tray/2000, establishing it as a reliable, straightforward model for water sample acquisition and monitoring. The microfluidic device was designed to have a 166.4 CFU/100 mL detection range and for detecting and quantifying bacteria below and above the EPA BAV value of 70 CFU/100 mL for *E. faecalis*, helping to reduce false negatives. Featuring self-loading capabilities upon immersion, the microfluidic device enables fast, user-friendly operation without requiring trained personnel. Additionally, the UV-LED/RGB system offers portable incubation and detection, effectively maintaining ideal bacterial growth conditions while monitoring bacteria growth and predicting contamination in a short amount of time.

The system was tested using various bacterial concentrations and benchmarked against the IDEXX Quanti-Tray/2000, demonstrating its ability to quantify bacteria growth over a range of concentrations. The characteristic sigmoidal growth curve’s slope becomes steeper, and the exponential phase begins earlier as the bacterial concentration increases. Positive wells were observed even at low concentrations, confirming the system’s detection capabilities at the EPA BAV standard for recreational water quality.

Notably, using normalized and first derivative RGBC signals as inputs in ML algorithms provided a method for accelerated bacterial detection. The results show that both the MLPNN and SVM exhibited strong classification performance, achieving a maximum precision of 1.00 across multiple sensors for both MLPNN and SVM. Importantly, SVM demonstrated the capability to predict the presence of bacteria in water samples within the first 30 min of incubation, achieving perfect metrics of 1.00 for accuracy, precision, recall, and F1 score for sensors 0, 1, and 4, with the lowest metric being 0.81 for sensor 3. While MLPNN achieved near-perfect metrics in most cases, SVM maintained robustness across various sensors in scenarios demanding rapid response times (30 min), delivering reliable performance using shorter lag phases, while MLPNN showed a decline in accuracy. This comparative analysis suggests that while MLPNN excels in stable conditions, SVM may be more suitable for dynamic environments requiring prompt detection. These findings underscore the effectiveness of the UV-LED/RGB system for MPN analysis combined with machine learning for rapid, low-cost, and portable bacteriological water quality assessments.

The system suffers from limitations, including its detection range and resolution and high confidence intervals (Figure 1e). Increasing the detection range and resolution and decreasing the confidence intervals requires introducing more wells into the microfluidic device, making it bigger, requiring more sensors, more energy for sample incubation, and more computational power for ML analysis. This would have drawbacks, such as higher energy requirements and reduced portability. Using only 10 mL in the microfluidic device makes it portable but negatively affects its accuracy while making it uncertain if the sample is representative of the environment being tested. The margins of error also increase with sample dilution, as required when analyzing saline water. Even with these drawbacks, the system demonstrates the ability to deliver bacteriological water quality results within 30 min in a low-cost, portable, and easy-to-use form factor, in stark contrast to traditional laboratory-based systems, which often require specialized equipment, laboratory space, and trained personnel.

## Figures and Tables

**Table 1:** Comparison between Quanti-tray/2000 benchmark and UV-LED/RGB system.

Pellet [CFU]	Quanti-Tray/2000 [CFU]	UV-LED/RGB System [CFU]
50–80	35.0	37.6
50–80	31.7	37.6
50–80	39.9	37.6
50–80	42.0	110.9
80–120	84.8	23.0
80–120	70.6	55.5
80–120	90.8	166.4
130–300	165.8	110.9
3000–7000	>2419.6	>166.4
50,000–150,000	>2419.6	>166.4

**Table 2:** Performance metrics of MLPNN and SVM algorithms across multiple sensors at varying lag phases for bacterial detection in water samples.

ML Algorithms	Time in Lag Phase [hours]	Evaluation Metrics	Sensors							
			0	1	2	3	4	5	6	7
MLPNN	3	Accuracy	0.99	1.00	0.99	0.81	0.94	0.96	0.93	0.91
		Precision	0.99	1.00	0.99	0.86	0.95	0.96	0.93	0.93
		Recall	0.99	1.00	0.99	0.81	0.94	0.96	0.93	0.91
		F1 Score	0.99	1.00	0.99	0.79	0.94	0.96	0.93	0.91
	2	Accuracy	0.98	1.00	0.99	0.81	0.97	0.94	0.95	0.96
		Precision	0.98	1.00	0.99	0.83	0.97	0.94	0.95	0.96
		Recall	0.98	1.00	0.99	0.81	0.97	0.94	0.95	0.96
		F1 Score	0.98	1.00	0.99	0.80	0.00	0.93	0.95	0.96
	1	Accuracy	0.95	1.00	0.97	0.79	1.00	0.96	0.92	0.92
		Precision	0.95	1.00	0.97	0.79	1.00	0.96	0.92	0.92
		Recall	0.95	1.00	0.97	0.79	1.00	0.96	0.92	0.92
		F1 Score	0.95	1.00	0.97	0.79	0.00	0.96	0.92	0.92
	0.5	Accuracy	0.93	0.99	0.96	0.77	1.00	0.92	0.77	0.72
		Precision	0.94	0.99	0.96	0.76	1.00	0.92	0.78	0.73
		Recall	0.93	0.99	0.96	0.77	1.00	0.92	0.77	0.72
		F1 Score	0.93	0.99	0.96	0.76	0.00	0.92	0.77	0.72
SVM	3	Accuracy	1.00	1.00	0.94	0.81	1.00	0.99	0.95	0.97
		Precision	1.00	1.00	0.94	0.85	1.00	0.99	0.95	0.97
		Recall	1.00	1.00	0.94	0.81	1.00	0.99	0.95	0.97
		F1 Score	1.00	1.00	0.94	0.79	1.00	0.99	0.95	0.97
	2	Accuracy	1.00	1.00	0.91	0.84	1.00	0.99	0.96	0.99
		Precision	1.00	1.00	0.92	0.87	1.00	0.99	0.96	0.99
		Recall	1.00	1.00	0.91	0.84	1.00	0.99	0.96	0.99
		F1 Score	1.00	1.00	0.91	0.83	1.00	0.99	0.96	0.99
	1	Accuracy	1.00	1.00	0.90	0.81	1.00	0.98	0.94	0.97
		Precision	1.00	1.00	0.90	0.84	1.00	0.98	0.95	0.97
		Recall	1.00	1.00	0.90	0.81	1.00	0.98	0.94	0.97
		F1 Score	1.00	1.00	0.90	0.79	1.00	0.98	0.94	0.97
	0.5	Accuracy	1.00	1.00	0.84	0.85	1.00	0.89	0.92	0.92
		Precision	1.00	1.00	0.84	0.85	1.00	0.90	0.92	0.93
		Recall	1.00	1.00	0.84	0.85	1.00	0.89	0.92	0.92
		F1 Score	1.00	1.00	0.84	0.85	1.00	0.87	0.92	0.92

## Data Availability

Data are contained within the article.

## Abbreviations

The following abbreviations are used in this manuscript:

MPN	Most probable number
CFU	Colony Forming Units
AI	Artificial intelligence
ML	Machine learning
MLPNN	Multilayer perceptron neural network
SVM	Support Vector Machine
RGB	Red–green–blue
UV/LED	Ultraviolet light-emitting diode
EPA	Environmental Protection Agency
pH	Potential of hydrogen
EC	Electrical conductivity
Turb	Turbidity
TDS	Total dissolved solids
TPh	Total phosphorus
TNit	Total nitrogen
DO	Dissolved oxygen
2012 RWQC	2012 Recreational Water Quality Criteria
BAV	Beach Action Value
MTF	Multitube fermentation
MF	Membrane filtration
RPA	Recombinase polymerase amplification
LFA	Lateral flow assay
RF	Random Forest
MLR	Multiple linear regression
WQI	Water quality index
TP	True positives
TN	True negatives
FP	False positives
FN	False negatives
ROC	Receiver operating characteristic
AUC	Areas under the curve
